# Comment on “Alpha-Lipoic Acid and Antioxidant Diet Help to Improve Endothelial Dysfunction in Adolescents with Type 1 Diabetes: A Pilot Trial”

**DOI:** 10.1155/2015/646095

**Published:** 2015-07-27

**Authors:** Hitesh Verma, Rajeev Garg

**Affiliations:** ^1^Overseas R&D Centre, Overseas HealthCare Pvt. Ltd., Phillaur, Punjab 144410, India; ^2^Department of Pharmaceutics, ASBASJSM College of Pharmacy, Bela, Ropar 140111, India

We read the research of Scaramuzza et al. [1], with great interest. The role of endothelial dysfunction in type 1 and type 2 diabetes is well evident and is related to various types of micro- and macrovascular complications associated with diabetes [2, 3]. In their study, Scaramuzza et al. depict the role of alpha-lipoic acid in alleviating the symptoms of endothelial dysfunction but the study seems to be associated with two major limitations. First and foremost limitation of study is the selection of appropriate dosage in the study protocol. The present study is conducted at the dose regimen of 400 mg twice daily (i.e., 800 mg/day). The rationale behind the selection of dose does not seem to be justified. The authors designed their hypothesis based on the results available in type 2 diabetic patients. Though the authors mentioned that in type 2 diabetes patients 600 mg/day dose is reported to produce significant benefits in endothelial dysfunction, still they used 800 mg/day regimen, which was not justified. Moreover, the authors did not include some very important references in their review of literature which were conducted for alpha-lipoic acid usage in alleviating endothelial dysfunction associated symptoms in type 1 diabetic patients. These trials were conducted at the dose of 600 mg/day and include adult population [4, 5]. These could serve as the basis for selection of appropriate dose as in our view the trial should have been conducted at a dose not more than 600 mg/day. Dose selection based on observation in type 2 diabetic patient seems to be inappropriate since endothelial dysfunction is more severe in case of type 2 diabetes which is often associated with various metabolic abnormalities (e.g., obesity, hypercholesterolemia, and hyperlipidemia) which is generally not the case in type 1 diabetic patients [3]. The second major limitation is the uncontrolled nature of the study; noncompliance among patients affects the study results. We appreciate the work of Scaramuzza et al., for starting the research of the possible role of alpha-lipoic acid in alleviating the endothelial dysfunction in young adolescent type 1 diabetic patients but future well-randomized, placebo controlled trials are required before coming on to some definite conclusion.
